# A Rare Case of Acinar Cell Cystadenoma in a 14-Year-Old Adolescent: A Case Report

**DOI:** 10.1089/crpc.2015.29009.nco

**Published:** 2016-01-01

**Authors:** Natalie Cosgrove, Joan DiPalma, Douglas Katz, Thomas Kowalski

**Affiliations:** ^1^ Division of Gastroenterology and Hepatology, Department of Internal Medicine, Thomas Jefferson University Hospital, Philadelphia, Pennsylvania.; ^2^ Division of Pediatric Gastroenterology, Department of Pediatrics, Nemours duPont, Philadelphia, Pennsylvania.; ^3^ Division of Pediatric General Surgery, Department of Surgery, Nemours duPont, Wilmington, Delaware.

**Keywords:** acinar cell cystadenoma, pancreas, pancreatic cyst

## Abstract

**Background:** Acinar cell cystadenoma is a rare pancreatic cyst that has been described in several case reports. This lesion may be incidental or asymptomatic, occurs predominately in females, and has a mean age of onset in the fourth decade.

**Case Presentation:** A previously healthy 14-year-old male presented with abdominal pain. He was found to have a pancreatic cystic lesion on ultrasound and cross-sectional imaging. His diagnosis remained uncertain despite additional analysis, including endoscopic ultrasound with fine-needle aspiration. The patient underwent successful laparoscopic excision for definitive diagnosis and management with an unremarkable postoperative course. He was diagnosed with a multilocular acinar cell cystadenoma.

**Conclusion:** Acinar cell cystadenoma is a rare pancreatic cyst whose true malignant potential is unknown. Although there are no formal recommendations for post-operative monitoring and the true risk of recurrence is unknown, we recommended every other year magnetic resonance imaging/magnetic resonance cholangiopancreatography for postresection surveillance for this patient due to the theoretical risk of recurrence with malignant transformation.

## Background

Incidental pancreatic cystic lesions are becoming increasingly common with more widespread use of cross-sectional imaging. Pancreatic acinar cell cystadenocarcinoma and acinar cell cystadenoma (ACA) account for <5% of these lesions. Acinar cell cystadenoma is a rare pancreatic cyst that has been described in several case reports.^1–3^ ACA typically arise from a background of normal pancreatic parenchyma and may be focal or diffuse in distribution. ACA may be unilocular or multilocular, with larger locules harboring internal septations. Unilocular ACA are predominately lined by well-differentiated acinar epithelium without atypia and scattered ductal cells, whereas patches of ductal epithelium within a background of acinar epithelium are more typical of multilocular ACA.^2^

Acinar cell cystadenoma occurs predominantly in females (61–88% female), with the age of diagnosis ranging from 9 to 66 years old and mean age of onset in the fourth decade.^1,2,4^ Cysts may be incidental or symptomatic. When symptomatic, the most common presentation is pain. We describe a case of this rare cystic lesion occurring in a healthy adolescent male presenting with abdominal pain.

## Case Report

**FIG. 1.**
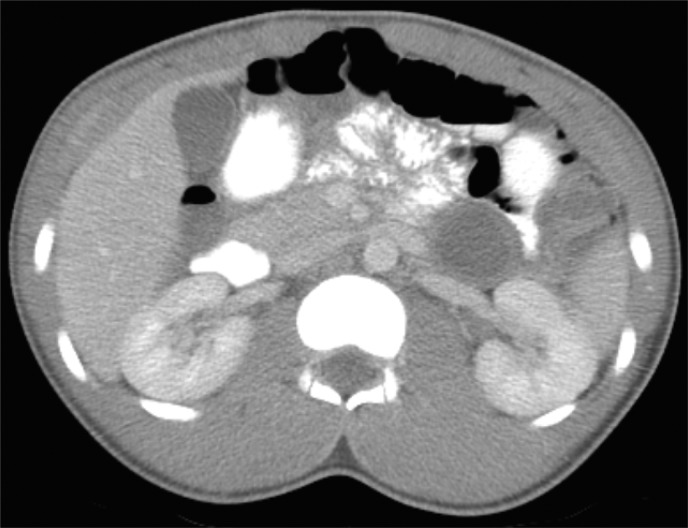
Computer tomographic scan of abdomen. Multiseptated cystic structure in the pancreatic tail (3.5 × 4 × 5 cm).

A previously healthy 14-year-old male presented to an emergency room with 3–4 days of diarrhea and abdominal pain in November 2014. Abdominal X-ray demonstrated dilated loops of bowel consistent with gastroenteritis. His serum aspartate transaminase was slightly elevated at 65 U/L (ULN 37); however, his remaining hepatic function panel, complete blood count, lipase, and amylase were normal. An abdominal ultrasound suggested a cystic structure medial to the left kidney with thickened internal septations. Despite repeating the abdominal ultrasound in multiple planes, it remained unclear whether the structure represented a distended fluid-filled loop of colon or a unique cystic lesion. Subsequent computerized axial tomography (Fig. 1) revealed a multiseptated cystic structure in the pancreatic tail measuring 3.5 × 4 × 5 cm with peripheral hyperdensities compatible with extrapancreatic calcifications. Several of the cystic components demonstrated thickened walls with mild enhancement. The pancreatic parenchyma and pancreatic duct appeared normal. No other pancreatic lesions were identified. Multiple mildly dilated fluid-filled loops of large and small bowel with air-fluid levels compatible with gastroenteritis were also noted, which were felt to be the likely etiology of his symptoms.

**FIG. 2.**
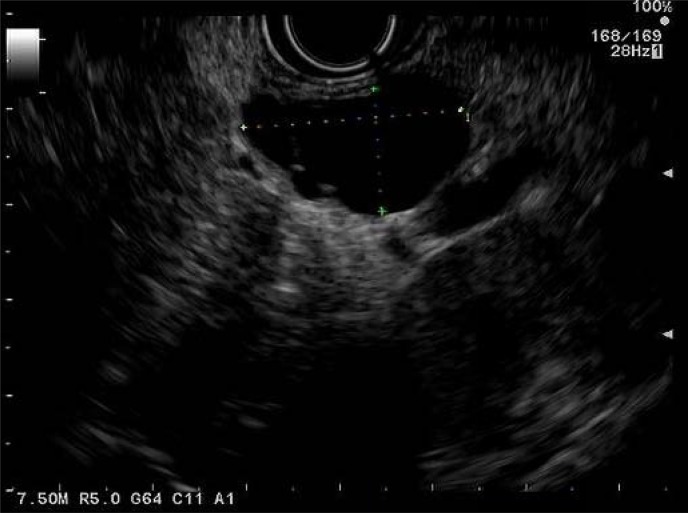
Endoscopic ultrasound image of a 2.3 × 1.3 cm anechoic lesion adjacent to the pancreatic tail.

The patient's symptoms resolved shortly following his emergency room visit, with normalization of his stools and resolution of his abdominal pain. After careful review of his images, his pancreatic lesion was felt to represent a cystic pancreatic mass and less likely a pseudocyst or a solid pseudopapillary tumor with cystic components. He was referred for an endoscopic ultrasound (EUS) to further characterize his lesion. An EUS (Fig. 2) performed in December 2014 noted a 2.2 × 2.2 cm thick-walled pancreatic tail lesion with a hypoechoic center and several adjacent anechoic lesions, the largest of which was 2.3 × 1.3 cm. The pancreas otherwise appeared normal. Fine needle aspiration of the largest anechoic lesion was performed using a 22G Expect^™^ BSCI needle. One milliliter of pink tinged nonviscous fluid was aspirated. Cyst fluid analysis revealed carcinoembryonic antigen 7.1 ng/mL and amylase 633 U/L. Aspirate cytology was nondiagnostic with rare small groups of mildly atypical epithelial cells, benign acinar cells, ductal cells, and fragments of fibrous tissue and debris. RedPath PathFinderTG^®^ integrated diagnosis was also indeterminate, due to lack of polymerase chain reaction amplifiability from low DNA quantity and poor DNA quality.

**FIG. 3.**
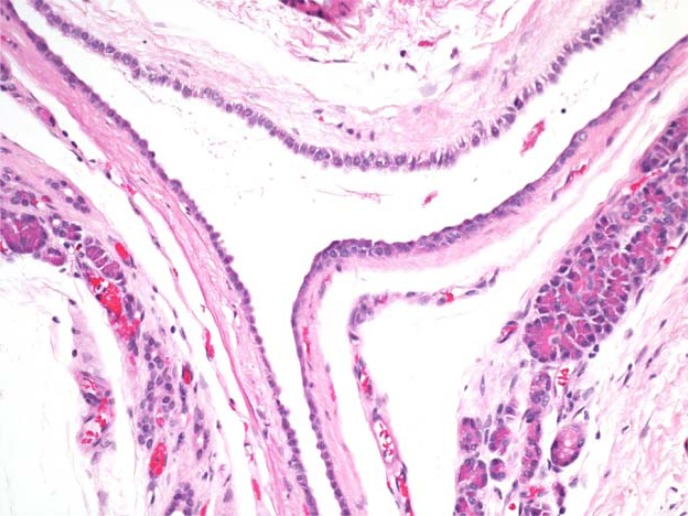
High-powered magnification of cysts lined by a single epithelial layer of cuboidal to columnar epithelium with focal acini.

The patient felt generally well and remained active in several sports, but continued to have episodes of recurrent abdominal pain. Repeat contrast-enhanced abdominal computed tomography in July 2015 demonstrated a persistent pancreatic cystic lesion measuring 2.6 × 3.1 × 1.8 cm, with prior imaging not available for comparison at that time. Although a definitive diagnosis had not been established, due to his recurrent pain and persistent lesion, the patient underwent laparoscopic excision in July 2015. The patient tolerated the procedure well. The lesion was easily dissected from the pancreas. Macroscopically, it was noted to have four cystic components, ranging in size from 0.8 × 0.7 × 0.4 to 2.6 × 1.6 × 1 cm. Each cyst contained a white smooth wall filled with white cloudy watery fluid. Microscopically, the cysts were lined by a single layer of cuboidal to columnar epithelium with focal acini, with one cyst demonstrating internal concretions (Fig. 3). No cytological atypia or mitoses were present. These features were consistent with a multilocular acinar cell cystadenoma.

## Discussion

We have described a case of a young adolescent male with a multilocular acinar cell cystadenoma who underwent successful cyst excision. To our knowledge, he is the second youngest patient to have been reported with this lesion.^5^ In addition, although nearly all previously reported cases of ACA resulted in extensive pancreatic resections, including pancreaticoduodenectomy, lateral pancreatectomy, and total pancreatectomy, we have described a case with successful cyst excision without pancreas resection. Postoperatively, we have recommended every other year magnetic resonance imaging/magnetic resonance cholangiopancreatography surveillance for this patient. Whether or not this is warranted or beneficial, especially given this patient's young age at diagnosis, is unclear.

It has been proposed that ACA originate from nonneoplastic acinar dilatation that expand into and incorporate ducts and ductules, eventually forming large cystic lesions as secretions accumulate. Ductal-to-acinar metaplasia has also been a theoretical explanation.^1^ More recently, however, pancreatic acinar cell cystadenoma has been described as an adenoma.^1^ Although it has been suggested that this lesion is a precursor to acinar cell cystadenocarcinoma, the true malignant potential of this lesion remains unclear.^1^ To our knowledge, no case report or case series has reported evidence of malignant transformation of ACA with up to 7.8-year follow-up, regardless of whether or not the lesion was completely resected.^1,2^ Although ACA lack atypia and mitotic activity that seems to favor a nonneoplastic lesion, the discovery of multiple chromosomal gains on comparative genomic hybridization that contain a few cancer-associated genes and genomic instability suggests the lesion is preneoplastic.^1^ In addition, a case of ACA with low-grade dysplasia was recently reported.^4^

We know from previous studies that ACA are likely to grow over time. In a case series of 10 patients, 4 of whom underwent sequential imaging, all four cysts showed interval enlargement over time with a mean increase of 1.5 cm for more than 2.9 years.^3^ No postoperative recurrences have been reported.^3^ Until the course of ACA is better established, however, we believe these lesions should be aggressively monitored, with strong consideration for surgical resection to reduce the theoretical risk of malignant transformation. Given the rarity of these cysts, future studies to support this will likely be limited.

## Abbreviations Used

ACA: acinar cell cystadenoma
EUS: endoscopic ultrasound
